# Sponges for Dentists’ Use

**Published:** 1883-02

**Authors:** 


Sponges for Dentists' Use.—Small velvet sponges are a great convenience in the operating room. They are often of great service in absorbing the saliva which threatens encroachment upon an operation in progress. If a bowl containing slightly moistened sponges of assorted sizes be kept within easy reaching distance they will be used much more frequently than one would imagine, and will soon become a necessity for the removal of stains from instruments and from the rubber-dam, as well as from the person of both patient and operator. Mouth napkins are not always protective, and the operative dentist cannot be too fastidious regarding cleanliness.

---

Intra-Uterine Vaccination.—Dr. Truzzi vaccinated a number of pregnant women during the last three months of gestation, with a view to determine the protection, if any, afforded to the child. The results were negative, as the children were all successfully vaccinated a few days after birth.—*Centralblatt fur Gynakologie.*

